# A Novel Introgression Line Library Derived from a Wild Melon Gives Insights into the Genetics of Melon Domestication, Uncovering New Genetic Variability Useful for Breeding

**DOI:** 10.3390/ijms241210099

**Published:** 2023-06-14

**Authors:** Manuel Campos, Maria José Gonzalo, Aurora Díaz, Belén Picó, Maria Luisa Gómez-Guillamón, Antonio José Monforte, Cristina Esteras

**Affiliations:** 1 Instituto de Biología Molecular y Celular de Plantas (IBMCP), Consejo Superior de Investigaciones Científicas (CSIC), Universitat Politècnica de València, Ingeniero Fausto Elio s/n, 46022 Valencia, Spain; macamve1@alumni.upv.es (M.C.); magonpa1@upvnet.upv.es (M.J.G.); adiazb@cita-aragon.es (A.D.); 2 Departamento de Ciencia Vegetal, Centro de Investigación y Tecnología Agroalimentaria de Aragón (CITA), Avda, Montañana 930, 50059 Zaragoza, Spain; 3 Instituto Agroalimentario de Aragón–IA2, Centro de Investigación y Tecnología Agroalimentaria de Aragón (CITA), Universidad de Zaragoza, 50013 Zaragoza, Spain; 4 Instituto de Conservación y Mejora de la Agrodiversidad Valenciana (COMAV-UPV), Universitat Politècnica de València, Camino de Vera s/n, 46022 Valencia, Spain; mpicosi@btc.upv.es; 5 Instituto de Hortofruticultura Subtropical y Mediterránea ‘La Mayora’ (IHSM, CSIC-UMA), Algarrobo-Costa, 29750 Málaga, Spain; guillamon@eelm.csic.es

**Keywords:** exotic germplasm, Piel de Sapo, fruit morphology, quality, QTL, transgressive segregation

## Abstract

A collection of 30 melon introgression lines (ILs) was developed from the wild accession Ames 24297 (TRI) into ‘Piel de Sapo’ (PS) genetic background. Each IL carried an average of 1.4 introgressions from TRI, and the introgressions represented 91.4% of the TRI genome. Twenty-two ILs, representing 75% of the TRI genome, were evaluated in greenhouse (Algarrobo and Meliana) and field (Alcàsser) trials, mainly to study traits related to domestication syndrome such as fruit weight (FW) and flesh content (FFP), as well as other fruit quality traits as fruit shape (FS), flesh firmness (FF), soluble solid concentration (SSC), rind color and abscission layer. The IL collection showed an impressive variation in size-related traits, with FW ranging from 800 to 4100 g, reflecting the strong effect of the wild genome on these traits. Most of the ILs produced smaller fruits compared with PS; however, unexpectedly, the IL TRI05-2 produced bigger fruits, likely due to new epistatic interacions with the PS genetic background. In contrast, the genotypic effect for FS was smaller, and few QTLs with notable effects were detected. Interestingly, variability was also observed for FFP, FF and SSC, rind color and abscission layer formation. Genes in these introgressions are candidates for having been involved in melon domestication and diversification as well. These results confirm that the TRI IL collection is a very powerful tool for mapping traits of agronomic interest in melon, allowing the confirmation of previously reported QTLs and the identification of new ones to better understand the domestication process of this crop.

## 1. Introduction

Melon (*Cucumis melo* L.) is one of the most important Cucurbitaceae crops, cultivated worldwide and with a steady production of over 26 million Tons in recent years (FAO, www.fao.org/faostat, accessed on 5 April 2023). A wide range of accessions including landraces, traditional cultivars, and modern hybrids—which exhibit an impressive diversity in plant and fruit characteristics, are suited to different culinary and consumer preferences and are adapted to several growing habitats [1]—are cultivated or stored in germplasm banks. This material has been developed since the domestication of wild melons around 4000 years ago [2].

Wild melons can still be found in Central Asia and Southern and Eastern Africa, displaying similar phenotypic characteristics such as small leaves, monoecious flowers, and round/oval small (20–50 g) fruit with very thin and inedible flesh surrounding small seeds [1]. Despite these similarities, wild African and Asian accessions are genetically distinct [3], and more closely related to cultivated landraces from their respective continents than to each other [4]. In fact, Endl et al. [5] proposed a taxonomic division of *C. melo* in two subspecies: *C. melo* subsp. *melo*, restricted to Asia, and *C. melo* subsp. *meloides*, restricted to Africa. The worldwide cultivated melons belong to subsp. *melo*, while subsp. *meloides* cultivars are only found in Sudan. Domestication events occurred independently in both subspecies, and Zhao et al. [6] suggested two independent events within subsp. *melo*: one event was the origin of worldwide cultivars (classified as subsp. *melo* by Zhao et al. [6]), and another event was the origin of cultivars grown specifically in East Asia (classified as subsp. *agrestis* by Zhao et al. [6]).

The genetic basis of crop domestication has been one of the hot topics in plant genetics over the last two decades [7]. This research included disciplines such as plant genetics, genomics, paleogenomics and archaeology. Domestication can be defined as the process of selection of individuals with characteristics on certain traits that fit human preferences that distinguish domesticated types from wild progenitors [8]. One approach to study the genetics of domestication is to investigate those traits, also referred as domestication syndrome traits, in experimental populations derived from crosses between domesticated and wild forms. Another approach is to identify selective sweeps between cultivated and wild relative genotypes, given that a reduction in genetic diversity in regions surrounding the domestication genes in the cultivated gene pool is expected. For the first approach, the definition of the domestication syndrome traits is crucial. In the case of melon, domestication traits include larger fruit, non-bitter taste, thicker flesh and leaves [1]. Up to now, the most thorough mapping study of melon domestication traits was carried out by Díaz et al. [9] in a cross between the Spanish ’Piel de Sapo’ (PS) variety and the wild accession Ames 24297 (*C. melo* subsp. *melo* f. agrestis [5]; TRI) from Pakistan, and stored in the North Central Regional Plant Introduction Station (Ames, IA, USA). Díaz et al. [9], in a F_2_ population derived from the cited cross, detected three fruit weight (FW) Quantitative Trait Loci (QTLs) in chromosomes 4, 6 and 8 where TRI alleles decreased the trait. The three QTLs drastically decreased FW (40–50%) when introduced into elite PS background, making them candidate loci to have been selected during domestication to increase FW. Regarding edible flesh content, two QTLs that decreased the trait were mapped on chromosomes 6 and 8. An epistatic QTL on chromosome 11 was also identified in a subsequent work [10]. Zhao et al. [6] also detected FW QTLs in the same regions of chromosomes 6 and 8, and additional QTLs on chromosomes 3, 5, and 7 in crosses between wild and cultivated melons. Lian et al. [11] found a FW QTL in the same region of chromosome 5 in an additional cross between wild and cultivated melons. Regarding the non-bitter taste, QTLs have been detected in chromosomes 9 and 11 in wild-x-cultivated crosses [6]. Zhao et al. [6] also studied selective sweeps between cultivated and wild melons, finding 148 and 185 putative selection sweeps in their defined *melo* and *agrestis* groups, respectively. Remarkably, all the FW and bitterness QTLs overlapped with domestication sweeps, reinforcing their possible role during domestication.

The analysis of wild relatives is not only useful to study the genetics of domestication, as these contain under-utilized genetic variations that could be used to improve modern cultivars. Wild species are currently being used in breeding programs, but mostly to introduce resistance to biotic stresses [12] and only scarcely for quality traits. In the case of melon, to our best knowledge, wild melons have not been exploited in modern breeding programs, and disease resistance genes currently used by breeders come from landraces and traditional cultivars [13]. Therefore, more attention should be paid to the wild melon genetic pool in order to uncover its potential. To fully exploit the potential of wild and un-adapted genetic variability, it is necessary to perform genetic experiments in advanced populations [14]. Thus, the fruits of the PS-x-TRI F_2_ are still very small compared with the cultivated PS (F_2_ FW mean ranged from 186 to 245 g, whereas PS mean ranges between 1300 to 2485 g), and epistatic interactions between TRI alleles from different loci may frequent [10,15]. An efficient way to manage exotic germplasm is the construction of genetic libraries of introgression lines (ILs). Each IL contains a unique introgression defined by molecular markers from a donor genotype (usually exotic or wild germplasm) in a uniform genetic background (usually an elite cultivar), and the introgressions introduced in a set of ILs ideally represents the entire genome of the donor genotype [16]. IL libraries have been extensively used in tomato [17,18,19,20], rice [21] and other crops to detect new genetic variability for complex agronomic traits and to introduce it into elite backgrounds. Several IL populations have been developed in melon using traditional or obsolete cultivars as donors. Interestingly, some of them share the PS genetic background, and the Korean ‘Songwan Charmi’ PI 161375 [22], the cantalupensis cultivar ‘Vedrantais’ [23] and the traditional Indian variety ‘Queen Anne’s Pocket Melon’ PI 273438 [24] have been used as donors. Additionally, ILs from the Indian accession PI 124112 have been developed in the PS background [25]. Additionally, ILs from the Japanese cultivar ‘Ginsen Makuwa’ PI 420176 [26] and PS [27] have been developed in the ‘Vedrantais’ background. The availability of IL collections in the same genetic backgrounds facilitates the study of the QTLs from diverse donors, as QTL-x-genetic background interactions do not occur, allowing for a direct comparison of the magnitude of the QTL effects, and also the study of QTL-x-QTL epistasis by simply crossing ILs with introgressions from different donors. A large number of QTLs involved in agronomically interesting traits have been identified within these IL collections ([28] and the references cited above. Additionally, disease resistance genes [29], QTLs involved in ripening [30] and fruit shape [31] have been cloned from those IL collections.

The objective of the present study was to construct an IL collection from the wild accession TRI into the PS background to shed light on melon domestication and to exploit the genetic variability present in the wild accession. The IL collection was evaluated in three independent agronomic trials to identify QTLs involved in external and internal fruit morphology, rind color and sugar content.

## 2. Results

### 2.1. Development of the Introgression Line (IL) Collection

**Figure 1:**
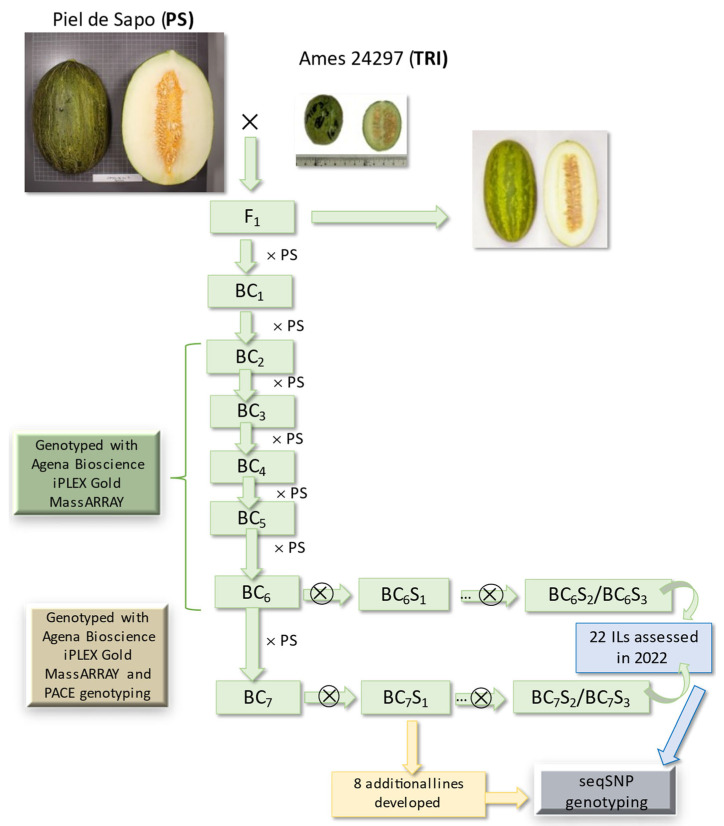
Breeding scheme followed for the development of the TRI introgression line (IL) population with the PS genetic background. Number of generations of backcross (BC) and selfing (S) is indicated, together with the genotyping platform employed for marker-assisted selection (MAS) to obtain the 22 ILs evaluated and the 8 ILs developed additionally.

The breeding scheme for the development of the IL collection is depicted on Figure 1. Initially, 15 backcross (BC) plants BC_1_ were backcrossed to PS, producing the 15 respective BC_2_ families. A total of 330 BC_2_ seedlings were genotyped with the 80-SNP (single nucleotide polymorphism) Agena Bioscience array (Table S1A), selecting 20 seedlings carrying an average of 5.9 introgressions/BC_2_ plant and representing the entire TRI genome. For the next step, 340 BC_3_ seedlings were genotyped with the 116-SNP Agena Bioscience array (Table S1A). A total of 26 seedlings were selected with an average of 6 introgressions/plant and 85.5% of the PS background genome (ranging from 71.9 to 97.1%), and representing 99.4% of the TRI genome.

**Figure 2:**
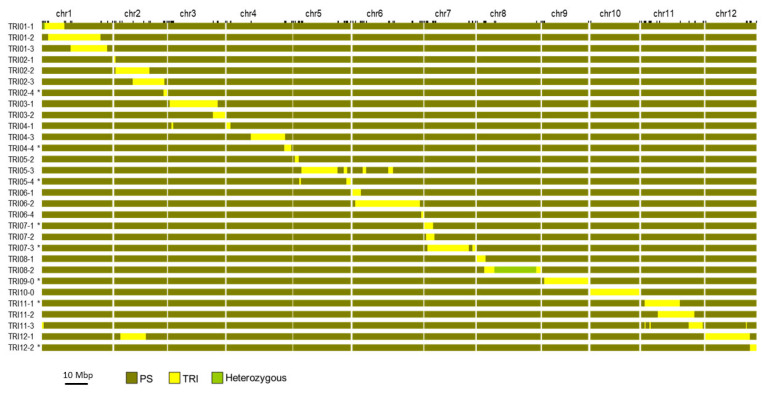
Graphical genotype of the 30 TRI introgression line (IL) collection. Each row represents an IL and the 12 melon chromosomes are represented by the columns. Asterisks indicate the eight additional ILs that were not evaluated in the 2022 trials.

Subsequent marker-assisted BC cycles were carried out with the 116-SNP array and PACE (PCR allele competitive extension) genotyping in final steps (Figure 1, Table S1A,B) to select plants with fewer introgressions to both recover the maximum percentage of the PS genome and represent the entire TRI genome. A first set of 22 ILs, most of them with a single TRI introgression, was selfed to obtain homozygous lines (BC_6_S_3_, BC_7_S_3_). This collection carried an average of 1.14 introgressions/line based on the genotyping with the 116-SNP Agena Bioscience array, with only 2 lines carrying 1 or 2 small introgressions apart from the target introgression. ILs were named according to the chromosome number where the TRI introgression was located, as well as the order from the start of the chromosome. The estimated percentage of the TRI genome represented in this IL set was 79.4%. The 22 ILs were further genotyped with 2590 SNPs using a seqSNP platform (Table S1C). The target introgressions were confirmed and defined more precisely, and additional small introgressions were detected (Table S2). Taking into account only introgressions of 0.1 Mbp or larger, the average number of introgressions per IL was 1.41 for the 22 ILs, with ILs TRI04-1 and TRI12-1 carrying two introgressions, TRI05-3 four introgressions and TRI11-3 five introgressions (Tables S2 and S3A). Figure 2 represents the graphic genotype of the TRI-IL collection, with a total coverage of the donor genome of 75.0%, ranging from 98.2% in chromosome 10 to 15.2% in chromosome 7 (Table S3B). Chromosome 9 was not represented in this first set of ILs. Some heterozygosity was observed in a fraction of the target introgressions carried by TRI01-1 and TRI08-2, with the other ILs completely fixed. The average length of the target introgressions (larger than 0.55 Mbp) was 11.56 Mbp with a maximum introgression length of 28.8 Mbp in chromosome 6 in TRI06-2.

An additional set of eight ILs was also generated to complete the coverage of the TRI genome and genotyped with seqSNP (Figure 2). Thus, the final IL collection consisted of 30 ILs, whose introgressions covered 91.4% of the TRI genome, ranging from 98.9% in chromosome 2 to 66.4% in chromosome 4 (Table S3B). In the 30-IL collection, the average number of introgressions per IL was 1.37, with the additional eight ILs carrying between 1 and 2 introgressions per IL (average of 1.25). Each chromosome is represented by an average of 3.4 introgressions coming from different ILs (2.6 introgressions/chromosome in the subset of 22-IL collection).

### 2.2. Phenotypic Characterization of Introgression Lines (ILs)

The selected set of 22 ILs was evaluated in three locations: Alcàsser, Meliana (Valencia) and Algarrobo (Málaga). Table 1 depicts the phenotypic values for the recurrent parent PS: fruit weight (FW; g), perimeter (FP; mm), length (FL; mm), width (FD; mm), shape (FS; calculated as the FL/FD ratio), fruit flesh proportion (FFP; calculated as the flesh area/total area ratio), flesh firmness (FF; kg/cm^2^) and soluble solid content (SSC; °Brix), which were compared among trials. The phenotypic behaviour of PS was very similar in all three locations, although statistically significant differences (*p* < 0.05) were detected for some traits. For instance, smaller and sweeter fruit were obtained in Algarrobo, whereas in Meliana, the fruit obtained were more elongated.

A two-way ANOVA (analysis of variance) was conducted to study the effects of the location, genotype and their interaction, considering the data coming from the three locations (3 L) (Table 2 and Table S4). The effect of the location and genotype was highly significant for all traits, except for FFP, where location effect was not significant. Since PS showed significant differences in Algarrobo with respect to the other two locations (Table 1), a two-way ANOVA was also performed with only Alcàsser and Meliana trials (2 L). In Algarrobo, there were some phytosanitary issues at the end of the season that might explain the differences obtained in that trial such as the smaller fruit. In this new ANOVA, the effect of the location was drastically reduced for all traits except for FS, and even showed a result that was not significant for FW. Traits related to the content of edible flesh, FFP and FA, also showed only significant genetic effects in this new analysis, with no significant effects of the location and the location-x-IL interaction. For all the traits, the IL effect was highly significant in both analyses, demonstrating the existence of strong genetic effects of the TRI introgressions. The interaction location-x-IL was only significant for FS in both analyses (3 L and 2 L).

Correlations between traits are depicted in Table S5. Size-related traits (FW, FP, FL, FD and FA) were highly correlated between themselves, with Pearson’s correlations I ranging from 0.64 to 0.99 (*p* < 0.001), and also with FFP (r = 0.26–0.48, at *p* < 0.001), i.e., the variation in FW was not only due to differences in the dimension of the fruit but also to the seed cavity. FS was highly correlated with FL (r = 0.61–0.73, *p* < 0.001), but not with FD, except in Algarrobo, where a low correlation (r = −0.21, *p* < 0.01) was presented. More unexpectedly, a moderate positive correlation was found between FS and FF.

**Figure 3:**
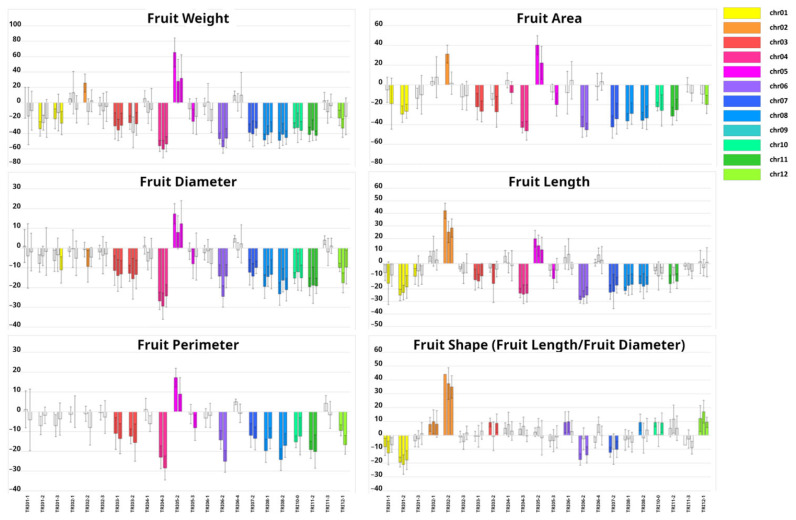
Variation in fruit morphology traits across the 22 TRI introgression lines (ILs). IL names are depicted on the bottom, where the bars represent the difference in percentage of the mean value of each IL with respect to the recurrent parent ‘Piel de Sapo’ (PS), with the standard deviation from the three locations (Alcàsser, Meliana and Algarrobo) in that order. The colored bars mean statistically significant differences (*p* < 0.05) between the IL and PS after a Dunnet’s test. The color of the bars indicate the chromosome where is located the target introgression according to the legend on the right.

**Figure 4:**
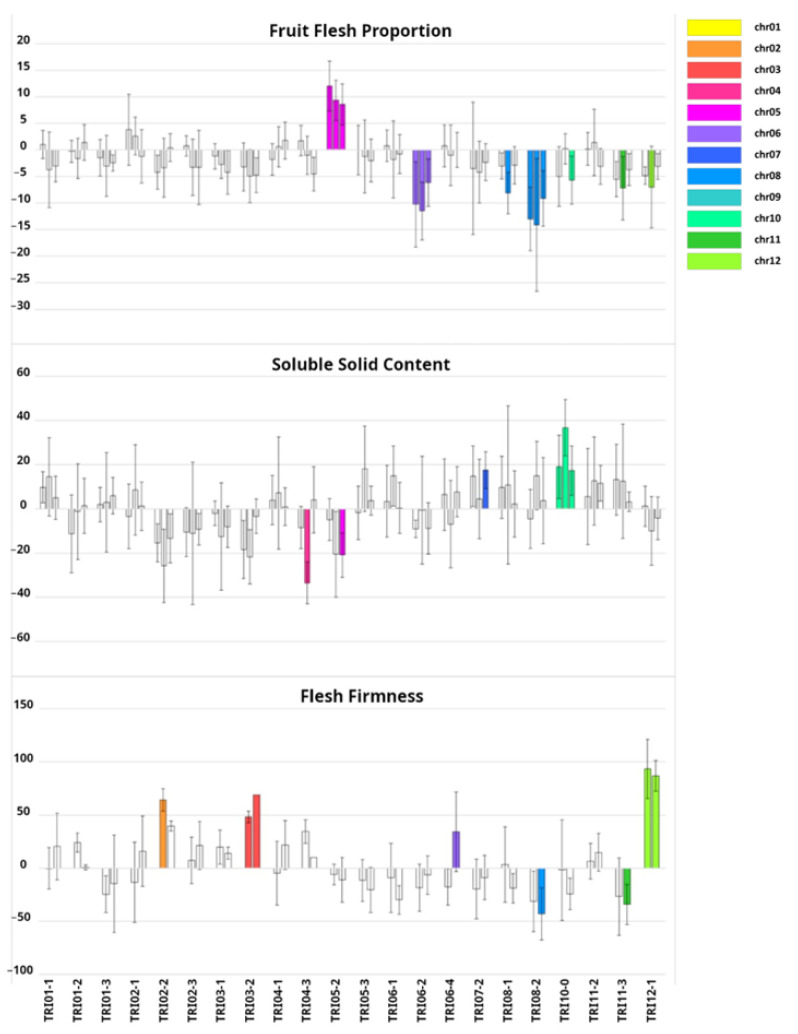
Variation in fruit quality traits among the 22 TRI introgression lines (ILs). IL names are depicted on the bottom, the bars represent the difference in percentage of the mean value of each IL with respect to the recurrent parent ‘Piel de Sapo’ (PS), with the standard deviation from the three locations (Alcàsser, Meliana and Algarrobo) in that order. For flesh firmness, the data were obtained only in Alcàsser and Meliana. The colored bars mean statistically significant differences (*p* < 0.05) between the IL and PS after a Dunnet’s test. The color of the bars indicate the chromosome where is located the target introgression according to the legend on the right.

Means and standard deviations for the traits in each of the 22 ILs are shown in Table S5. Figure 3 and Figure 4 depict the IL means as a percentage with respect to the PS control and the statistical significance of the comparison between ILs and PS with the Dunnett’s test. An IL was considered to have a significant effect on a trait only when the effect was significant at least in two locations. Representative fruit from each IL is shown in Figure S1.

**Figure 5:**
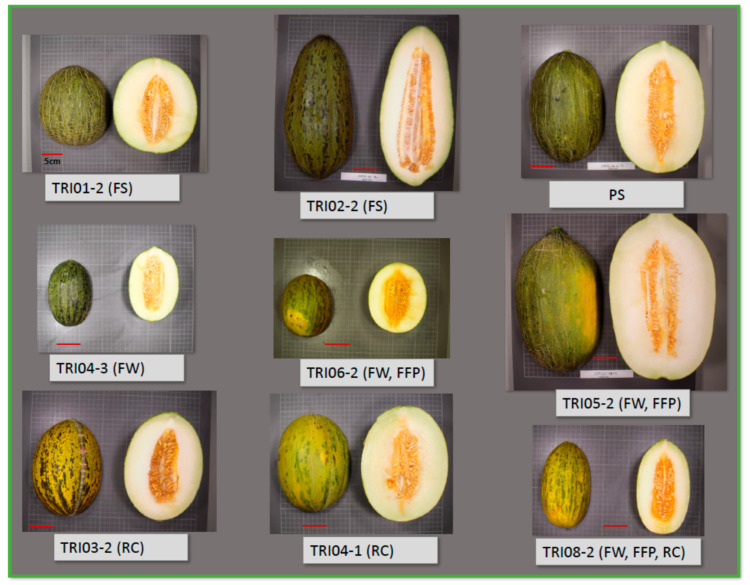
Pictures of representative fruit for ILs showing the larger effects in fruit weight (FW), fruit shape (FS), fruit flesh proportion (FFP) and rind color (RC). The pictures are at the same scale; the red bar represents 5 cm.

High variability was found across ILs in fruit size-related traits (FW, FP, FD, FL and FA). For instance, FW, as the most representative trait for fruit size, ranged from +42% (TRI05-2) to −57% (TRI04-3) compared to PS (Figure 3, Table S6). TRI05-2 was the only IL that increased FW, while 11 ILs displayed significant FW reduction, with TRI04-3, TRI06-2, TRI08-2 and TRI08-1 exhibiting the most striking reductions (−57%, −50%, −45%, and −43%, respectively) (Figure 5). The rest of the fruit size-related traits had a variation pattern very similar to FW (Figure 3).

Variability was also found between ILs for FS, although with a lower range. The most significant effect was observed in TRI02-2, which produced fruit that were 39% more elongated than PS (Figure 3 and Figure 5, Table S6), and carried the TRI introgression in the chromosome 2 region including the *andromonoecious* gene (*a*) whose wild allele induces monoecy. The mean FS of the rest of the ILs as compared with PS (Figure 3) ranged between +13% (slightly more elongated: TRI02-1, TRI03-2, TRI06-1, TRI10-0 and TRI12-1,) and −19% (almost round: TRI01-1, TRI01-2, TRI06-2, TRI07-2). The relationship between FS and its components, FL and FD, could be divided in three groups: (1) FS variation mostly due to variation in FL (TRI01-2, TRI02-2, TR06-2); (2) FS variation mostly due to variation in FD (TRI03-2, TRI10-0 and TRI12-1); (3) FS variation could not be clearly related either to variation in FL or in FD (TRI01-1, TRI02-1 and TRI06-1).

Variation in other important fruit quality-related traits was also observed (Figure 4, Table S6). TRI05-2 showed >10% of FFP as compared to PS, while TRI08-2 and TRI06-2 displayed a lower FFP, −12% and −9% with respect to PS, respectively (Figure 5). With regard to SSC, only TRI10-0 was significantly different from PS, exhibiting a 24% increase (Figure 4). Finally, FF, related to ripening, increased (firmer fruit) in TRI12-1 and TRI03-2 (+90% and +59%, respectively) as compared to PS.

Changes in rind color (RC) or the presence of the abscission layer (AL) were evaluated visually (Table 3). Fruit from IL TRI03-2 had a characteristic yellow background in the rind which was observed in the three locations, while a paler background with respect to PS was observed in both TRI04-1 and TRI08-2 (Table 3, Figure 5). Fruit from TRI08-1 and TRI08-2 showed an AL. This trait was not evaluated in fruit harvested in Algarrobo.

## 3. Discussion

### 3.1. Development of the Introgression Line (IL) Collection

In melon, several IL populations have been developed using the cultivar ‘Piel de Sapo’ [22,23,24] or the *cantalupensis* cultivar ‘Vedrantais’ [26,27] as the recurrent parents. So far, a wild melon as TRI has not yet been used as the donor. In the current work, we obtained the TRI-IL collection after 8 to 10 generations of MAS, a few more than in the previous melon collections (between 4 and 7) and in other crops such as tomato (5 to 7; [20]). Other works report a similar number of generations, such as 7 to 10 in rice [32] or 9 in tomato [33]. Fast and efficient development of IL collections can be achieved by performing MAS on a high number of seedlings using fast genotyping techniques capable of genotyping an adequate number of markers distributed throughout the genome [20]. Despite using a fast low-density SNP assay, we could not speed up IL development as in other studies [20].

In this work, we obtained a first set of 22 ILs that covered most of the TRI genome except some genomic regions mainly on chromosomes 4, 5, 7 and 9. We observed (although we did not do any specific experiments) that in every generation, plants carrying introgressions in those chromosomes showed low fruit set or seed germination, or only plants with several additional introgressions produced seed properly. Barrantes et al. [20], in their IL tomato library developed with a wild relative as donor, also reported similar problems of vigour associated with two genomic regions, though this was also due to a strong segregation distortion toward wild alleles probably caused by the presence of a “*Gamete eliminator*” locus (*Ge*). To assess if a similar phenomenon could occur in our population, we re-analyzed the allele segregation in the F_2_ PS-x-TRI from Díaz et al. [9]; however, no strong segregation distortion toward PS (which would explain the loss of some regions of TRI genome during IL development) was observed. Apart from factors related to vigour or fertility, some diseases and pests affected the plants during the IL development program, and the different susceptibilities to them could have also reduced the fertility of some plants. Additionally, some genomic regions such as regions in chromosomes 1 and 8 still remain heterozygous in the ILs, and others required several generations to be fixed. During the development of BC_2_S_3_ lines from a cross between the wild *Solanum habrochaites* by S. Knapp and D.M. Spooner and the processing tomato cultivar ‘E6203′, Monforte and Tanksley [18] also observed a higher level of heterozygosity than expected in several genome regions.

The final TRI-IL collection (30 ILs) presented herein represents around 91% of the TRI genome, with non-covered gaps mainly in chromosomes 4, 5 and 7 (Table S3B), similar to the 98% of the ‘Vedrantais’ genome representation in the 36 ILs in the PS background [23]. Other melon IL collections with the ‘Vedrantais’ genetic had a coverage ranging from 95 to 99% with 27 and 34 ILs, respectively [26,27]. Taking into account the additional difficulties associated to the introgression of wild genomes in cultivated types, the percentage of coverage obtained can be considered quite high. The lack of a total representation of donor genomes when developing IL populations is common due to several reasons such as a small initial population size, a small set of markers employed for MAS or vigour-related problems [20].

The average target introgression length was 11.2 Mbp in the 30-IL collection. Similar average sizes were reported for introgressions in previous melon IL collections ranging from 8.4 to 18.2 Mbp [23,24,26,27]. The average number of introgressions per IL in the TRI collection was similar to previous studies with other melon ILs, in which values ranged from 1 to 1.3 [23,26,27], except of the average 3.2 introgressions per IL reported by Castro et al. [24].

The graphical genotype of TRI06-2 and TRI06-4 was compatible with the presence of an inversion between positions 36,111,486 and 30,655,431 of chromosome 6 (Table S2) when the SNPs were mapped to the genome assembly v4.0 [34]. Pereira et al. [35] and Santo Domingo et al. [23] also found this possible inversion in populations derived from the cross PS-x-‘Vedrantais’, which suggests the need for a re-evaluation of the genome assembly in that region.

### 3.2. Genetic Dissection of Agronomic Traits with the Introgression Line (IL) Collection

The ANOVA indicated a highly significant IL effect on all studied traits, although the extent of the IL and location effect differed among traits. One striking observation was that fruit size traits (FW, FFP) showed a lower location effect (especially when comparing 2 locations) than FS, while usually FW QTLs show less consistent effects than FS QTLs [36]. In fact, 12 TRI ILs showed very consistent and strong effects (>30% as compared with PS) on FW, while most of the 10 ILs with significant effects on FS showed weak effects (~10%). Barrantes et al. [37] also found stronger and more consistent effects in FW QTLs than in FS QTLs in the tomato interspecific IL population *Solanum lycopersicum*-x-*Solanum pimpinellifolium*. Previous melon mapping populations were derived from crosses between cultivars, where high genetic variability for FS between parents could be expected as a consequence of cultivar diversification, whereas in the current IL population, the genetic variability for fruit size traits between the two parents was more important than for fruit shape traits, as size traits have been key during domestication, while shape traits are more related to diversification [1].

TRI alleles usually decreased fruit quality traits such FW, FFP, SSC and FF, but some ILs increased the traits. For example, TRI05-2 increased both FW and FFP, TRI10-0 consistently increased SSC, and TRI03-2 and TRI12-1 increased FF, demonstrating a transgressive segregation for these traits. Other unexpected phenotypes were the yellow background rind color in TRI03-2, and the presence of AL in most fruit from TRI08-1 and TRI08-2. The emergence of transgressive phenotypes in IL collections is very well documented [16], and it may be explained by the new allelic combinations or epistatic interactions with the receptor genetic background. The current results demonstrate that the TRI-IL collection can be also a useful source for improving melon fruit quality.

The genetic control of melon fruit morphology has been extensively studied in previous works [15,38]. Every QTL detected in the current report has been detected in a previous experimental population (Table S7). Among those previous works, some of them may be highlighted. Díaz et al. [9], using an F_2_ population derived from the same PS-x-TRI cross, identified three FW QTLs in the genomic regions covered by the ILs that also showed FW reduction: TRI04-3, TRI06-2 and TRI08-2. Three QTLs were also detected for FFP (PA in the previous work) that mapped in the TRI05-2, TRI06-2 and TRI08-2 introgressions. Riahi et al. [10] attempted to fine-map the FFP QTL on TRI06-2, but the results were inconsistent due to an epistatic interaction with a region in chromosome 11. In the current report, we have not found any consistent effects on FFP in any of the chromosome 11 ILs, confirming that there is not any additive QTL in that region. The fact that FFP effects on TRI06-2 were detected in the current research may be due to the higher statistical power of our current design, even though the TRI alleles at the epistatic locus on chromosome 11 were not present in TRI06-2. Díaz et al. [9] reported FS QTLs in chromosomes 2, 4 and 6. The QTL in chromosome 2 co-mapped with the *andromonoecious* gene [39], which induces fruit elongation [31]. Unlike the other ILs which are andromonoecious such as PS, TRI02-2 is monoecious and produced very elongated fruit (Figure 5 and Figure S1). The FS QTL in chromosome 6 overlapped with the TRI06-2 introgression (the IL that produced round fruit; Figure 5 and Figure S1), whereas ILs with introgression on chromosome 4 did not show any effect on FS. Argyris et al. [40] also reported QTLs for SSC on chromosomes 3 and 5, but they could not be validated in the current experiments. In general, a large proportion of QTLs detected in previous works from populations derived from the same cross were validated in the TRI-IL collection, and new QTLs were detected (i. e., TRI01-1, TRI01-2, TRI03-2, TRI06-1, TRI07-2, TRI10-0, TRI12-1 for FS; TRI01-3; TRI03-1, TRI03-2, TRI05-2, TRI07-2, TRI10-0, TR11-2 and TRI12-1 for FW; and TRI10-0 for SSC).

Zhao et al. [6] found FW QTLs in chromosomes 7 and 8 in a wild-x-cultivated agrestis cross compatible with the effects found in TRI07-2 and TRI08-2. Interestingly, both genomic regions showed selection signals for domestication, reinforcing those loci as strong candidates to have been involved in the melon domestication process. Lian et al. [11] also studied an F_2_ from another wild-x-cultivated agrestis cross, finding a FW QTL in chromosome 5, but the position was not covered by the TRI05-2 introgression, which might reflect genetic variability for FW loci among wild melons.

Strictly speaking, a domestication locus should not be variable within the cultivated gene pool, so it could not be mapped in crosses between cultivated accessions. This criterion may not be entirely valid for melon, as genetic exchange between cultivated and wild melons still occurs today in India, which is the primary centre of diversity [4], not only with landraces [41], but also with commercial cultivars [42]. Therefore, there is a continuous gradient between wild and cultivated melons [41]. Nevertheless, QTLs involved in FW previously described as consensus QTLs or detected frequently in different populations, mapped in regions covered by the introgressions present in TRI01-3, TR03-1, TRI03-2, TRI04-3, TRI05-2, TRI06-2, TRI07-2, TRI08-1, TRI08-2, TRI10-0, TRI11-2, TRI12-1 (Table S7, [15,23,27,35,38,43]). Therefore, it is not clear which of these loci could have been involved in melon domestication. With respect to FFP, very few studies have investigated this trait previously (e. g. Perpiñá et al. [26]). TRI06-2 and TRI08-2 consistently decreased FFP, and both ILs decreased FW, whereas TRI05-2 increased both FW and FFP, as commented above. Currently, we can not elucidate if these phenotypes are due to pleiotropy or linkage. Nonetheless, the colocation of genomic regions controlling both traits makes them good candidate regions for harboring domestication genes. Finally, TRI04-3 showed the highest effect on FW reduction, making this introgression another candidate to study domestication genes.

The analysis of the TRI-IL collection can also provide some insights into the biological and genetic basis of FW. Thus, all the ILs that decreased/increased FW showed a concomitant decrease/increase in FD. On the other hand, most of those ILs also showed a decrease/increase in FL, except for TRI03-2, TRI10-0 and TRI12-1, suggesting two mechanisms affecting FW, the first one affecting both fruit dimensions, and the second one affecting only FD. Regarding candidate genes, even though the mapping resolution of the TRI-IL collection was low, some interesting patterns were observed based on previously defined genes involved in fruit morphology [15,38]. Candidate genes previously reported for FW and FS (reviewed by Pan et al. [38]) were found in the regions spanned by TRI introgressions (Table S8). For instance, TRI03-1, TRI04-3, TRI06-2, TRI08-1, TRI8-2 and TRI10-0 introgressions contain members of the *Cell Number Regulator* (*CNR*) gene family, TRI03-1 and TRI10-0 members of the *CYP78A10*, and TRI05-2 a homologue of the *Cellular Size Regulator* (*CSR*). On the contrary, no obvious candidate genes were located in the introgressions of TRI01-3, TRI11-2 and TRI12-1, which may reflect the novel genes involved in FW.

Regarding diversification traits, the introgressions of the ILs that showed significant variations on FS overlapped with known consensus QTLs [15,38]. TRI02-2 was monoic and produced elongated fruit as expected; however, interestingly, TR02-1, andromonoecious with a small introgression at the beginning of chromosome 2 near the *a* locus, also produced elongated fruit, although with a lower effect than TRI02-2. This result indicates that there is a FS QTL linked to *a*, so the elongation observed when *a* is introduced into andromonoecious cultivars could be due not only to the pleiotropic effects of *a*, but also by that linked QTL. Castro et al. [24], in their ILs derived from two andromonoecious parentals, suggested the presence of other QTL different from the *a* gene involved in FL close to that genomic region. In addition, two different mechanisms modifying FS can be observed in this collection. The FS variation in TRI01-2, TRI02-2, TRI06-2 and TRI07-2 can be related to FL variation; however, FS variation in TRI03-2, TRI10-0 and TRI12-1 respond to changes in FD. Members of the *SUN*, *OFP* and *TRM* gene families have been demonstrated to be involved in fruit morphology in different species [15,44]. We found members of those gene families in the introgressions of most ILs altering FS, except for TRI01-1, TRI01-2 and TRI02-1, where no members of these gene families have been identified (Table S8).

With respect to SSC, the only IL that showed consistent effects was TRI10-0, which increased it. This result was unexpected as TRI does not develop edible flesh, and suggests that genes for sugar accumulation can be uncovered and exploited from wild types in melon. Perpiñá et al. (Table S7) [26] also found a consistent increase in SSC in the IL MAK10-1 that contained an introgression in chromosome 10 from the Japanese cultivar ‘Ginsen Makuwa’ into the ‘Vedrantais’ genetic background. This increase was attributed to the longer ripening time of MAK10-1 fruit as compared with the ‘Vedrantais’ fruit, which allowed for the accumulation of more sugars as the fruit could be maintained in the plant for a longer period of time due to the delayed development of the abscission layer. In the current work, TRI10-0 fruit were harvested at the same time as the other ILs, so the time the fruit was attached to the plant was not a factor involved in the increase in SSC. Finally, AL was observed in TRI08-1 and TRI08-2, with the TRI08-2 introgression compatible with the position of the major fruit ripening QTL *ETHQV8.1* defined by Pereira et al. [45] in a PS-x-‘Vedrantais’ cross. Given the length of the introgression, it is not possible to assess if it is the same QTL in both works. Nevertheless, the effects on TRI08-1 and TRI08-2 were much lower than those reported for *ETHQV8.1*, so it could be a weak allele. In addition, in TRI08-2, we observed a pale background in the rind in comparison to PS. Finally, TRI03-2 and TRI12-1 showed a high FF, a characteristic that is interesting for melon breeding. Further research is necessary to elucidate if that increase in FF is related to a delay of ripening or to flesh matrix changes. Interestingly, TRI03-2 also showed a change in rind color, showing a yellow rind background. Changes in color rind have been related to the ripening process, and Monforte et al. [46] already reported a QTL in chromosome 10, where the candidate gene *CmKFB*, responsible for the yellow phenotype, was later reported [47]. However, no QTL in chromosome 3 had previously been identified as involved in this trait to date.

As a summary, the current TRI-IL collection has been proven to be useful for dissecting the genetics of melon fruit morphology, especially related to domestication but also with diversification. Additionally, alleles from the wild TRI of interest for breeding (increase in FFP, SSC and FF) were identified. This collection provides additional information to the previous IL collections developed in the PS background. This group of IL collections could be defined as a Meta-IL collection, integrating five different donors from different horticultural groups [1] and country of origin: PI 161375 (chinensis, Republic of Korea [22]), PI 124112 (momordica, India [25]), PI 273438 (dudaim, Asian, not specified [24]), ‘Vedrantais’ (cantalupensis, France [23]) and the current TRI (wild f. agrestis, Pakistan). These donor genotypes represent genetically distinct melon groups [4], covering different allelic diversity in this species: from wild (TRI) to modern (‘Vedrantais’) cultivars and through ornamental (PI 273438) and old landraces incorporating several disease resistance genes (PI 161375, PI 124112).

## 4. Materials and Methods

### 4.1. Plant Material and Introgression Line (IL) Development

The parents of the IL population were the wild accession Ames 24297 (TRI) from Pakistan, obtained from the North Central Regional Plant Introduction Station as donor parental, and the Spanish cultivar ‘Piel de Sapo’ (PS) belonging to the horticultural group ibericus) [1] as the recurrent parent. PS is the most important melon cultivar type in Spain from an economical perspective. Typically, PS fruit are large (>2 kg), oval (FS = 1.3), very sweet (SSC > 12°), non-climacteric with large seeds and andromonoecious. On the other hand, TRI fruit are small (<50 g), with non-edible flesh, small seeds and momoecious [9]. The breeding scheme used to develop the IL collection is shown in Figure 1. The F_1_ hybrid from the cross PS-x-TRI was backcrossed with PS to obtain BC_1_ seeds. Several BC cycles were carried out, and seedlings were genotyped from each generation with appropriate markers (see below) to select those with the maximum content of the PS genome but covering the full TRI genome. A first set of selected BC_6_ and BC_7_ plants with one or two introgressions in heterozygosis were selected to obtain the first set of homozygous ILs after three self-pollination cycles (BC_6_S_3_, BC_7_S_3_). This IL set was used in the 2022 phenotyping assays (see below). An additional set of ILs (8 ILs) were developed later to increase TRI genome representation in the collection.

The cultivation of all generations mentioned above was carried out at greenhouse facilities at the Polytechnic University of Valencia (Valencia, Spain) and the Fundación Cajamar in Paiporta (Valencia, Spain).

### 4.2. Genotyping Methods

Genomic DNA was extracted from leaf samples as they were in Doyle and Doyle [48] with minor modifications [9]. The final concentration was adjusted to 10 ng/μL (Agena Bioscience array and PACE 2.0).

Seedling genotyping was carried out with the Agena Bioscience platform iPLEX^®^ Gold MassARRAY system (Agena Bioscience GmbH, Hamburg, Germany) at the Epigenetics and Genotyping laboratory (Central Research Unit of the Faculty of Medicine, UCIM, of the University of Valencia, Spain). For this purpose, a set of 80 SNPs was employed in the BC_2_ generation, and subsequently extended to 116 SNPs for the following generations (Table S1A). This new 116-SNP set was designed with markers that were evenly distributed throughout the melon genome (7 to 12 SNPs/chromosome), as previously defined by Garcia–Mas et al. [49], Esteras et al. [3] and validated by Díaz et al. [9] and from a previous GBS (genotyping by sequencing) dataset [4]. In advanced BC and selfing cycles, SNP genotyping was performed by PCR allele competitive extension (PACE) with the PACE^®^ 2.0 Genotyping Master Mix (3CR Bioscience, Harlow, UK) following the 3CR Bioscience recommendations (Table S1B).

The IL collection was finally genotyped using seqSNP technology (targeted genotyping by sequencing), a massively parallel SNP screening based on NGS (Biosearch Technologies, LGC Genomics GmbH, Berlin, Germany). Flanking sequences of selected markers according to previous GBS data [4] and resequencing of TRI [50] were submitted to LGC for primer design and synthesis (Table S1C). Leaf samples collected with the BioArk plant collection kit were sent to the company where extraction, library preparation, 75bp single read Illumina NextSeq 500/550 v2 sequencing and standard bioinformatic analyses were performed. Bowtie2 v2.2.3 was used for the mapping of previously trimmed and quality-filtered reads (Phred quality score ≥ 30, final read length > 65 bp) to the melon reference genome (assembly v.4; [34]), and Freebayes v1.2.0 for SNP calling (vcf file), where the read threshold for genotype calls was established in 8.

GGT v2.0 software [51] was employed to generate the graphical genotype of the collection, previously transformed using the software TASSEL v5.0 [52] to ABH format from the original vcf file.

According to the seqSNP genotyping data, the size of the introgressions was calculated following the assumption that introgresision extremes were the same as the first or last genotyped SNP with the TRI allele. In the first generations with a small set of SNPs and thus larger distances among them, the assumption was that the recombination point was found in the intermediate position between two boundary genotyped SNPs (TRI allele and PS allele).

### 4.3. Phenotypic Characterization of the Introgression Line (IL) Collection

Twenty-two ILs were evaluated in three trials during the 2022 spring–summer season (March to mid-July). Meliana (Valencia, Spain) and Algarrobo (IHSM La Mayora, Málaga, Spain) trials were conducted under plastic greenhouse with integrated pest control, whereas Alcàsser (Valencia, Spain) trial was conducted in an open field under organic farming management.

The Meliana and Algarrobo trials followed a randomized block design with five blocks with two single plant replicates of each IL and ten replicates of PS. The Alcàsser trial consisted of five blocks with a single plot replicate of three plants for each IL, and five plots for PS. In the three assays, all the plants were open-pollinated with bumblebees. When more than one fruit was obtained (mostly in Alcàsser; in Meliana only one fruit per plant was retained), the most representative fruit were selected for phenotyping. Plants were grown on soil with fertigation in the three trials. Pruning to control vegetative growth and flowering was frequently performed.

The traits evaluated, related to fruit morphology, were the following: fruit weight (FW; g); perimeter (FP; mm); length (FL; mm); width (FD; mm); shape calculated as the FL/FD ratio (FS); fruit area (FA; mm^2^) measured on digital images obtained with a scanner and analyzed with the Ilastik pixel and object classification software (Version 1.4.0rc6) [53]; and fruit flesh proportion as the flesh area/total area ratio (FFP), measured on digital images obtained with a scanner and also analyzed with Ilastik pixel and object classification. Regarding fruit quality attributes, flesh firmness (FF; kg/cm^2^) was measured at 4 points in the equatorial region of the mesocarp using a pressure tester FT327 with a plunger diameter of 8 mm (Alfonsine, Italy), and soluble solid content (SSC) was measured as °Brix from juice from the equatorial region of mesocarp using a Pocket refractometer (PAL-α, Atago CO., LTD, Tokyo, Japan). The presence of the abscission layer (AL) and the rind color (RC) were visually assessed.

### 4.4. Statistical Analyses

A two-way ANOVA was performed with Jamovi statistical package v2.3 to estimate the IL and location effects and their interactions. A correlation matrix was generated with Pearson’s coefficient with the same software. Correlations with *p* < 0.01 were considered significant. The statistical package JMP 12 v5.1 (SAS Institute, Cary, NC, USA) was used to compare IL means with the PS mean with Dunnett’s contrast (*p* < 0.05).

## Figures and Tables

**Table 1:** Mean and standard deviation of the recurrent PS parent in the three locations assayed (Alcàsser, Meliana and Algarrobo) for the quantitative traits assessed. Different letters (a, b, c) indicate statistically significant different means (*p* < 0.05).

Trait ^1^	Alcàsser	Meliana	Algarrobo
FW (g)	2514.48 ± 324.73 ^a^	2637.36 ± 508.29 ^a^	1752.68 ± 459.23 ^b^
FP (mm)	483.56 ± 23.18 ^a^	473.68 ± 31.47 ^a^	n.d. ^2^
FS	1.39 ± 0.06 ^a^	1.52 ± 0.13 ^b^	1.41 ± 0.12 ^a^
FL (mm)	215.08 ± 11.32 ^a^	231.08 ± 19.98 ^b^	185.16 ± 14.97 ^c^
FD (mm)	154.72 ± 7.19 ^a^	152.36 ± 9.99 ^a^	132.74 ± 12.72 ^b^
FA (cm^2^)	263.16 ± 22.80 ^a^	286.22 ± 38.39 ^b^	n.d.
FFP (%)	70.32 ± 3.00 ^a^	70.42 ± 3.21 ^a^	70.88 ± 3.53 ^a^
SSC (ºBrix)	12.24 ± 1.30 ^a^	10.40 ± 2.72 ^b^	13.92 ± 1.60 ^c^
FF (kg/cm^2^)	2.12 ± 0.43 ^a^	2.55 ± 0.85 ^b^	n.d.

^1^ Traits analyzed: FW (fruit weight), FP (fruit perimeter), FS (fruit shape, ratio length/diameter), FL (fruit length), FD (fruit diameter), FA (fruit area), FFP (fruit flesh proportion), SSC (soluble solid content) and FF (fruit firmness). ^2^ n.d.: no data.

**Table 2:** Two-way ANOVA to study the effect of location (L), introgression line (IL) and L-x-IL interaction for each trait analyzed taking into consideration data from three locations (Alcàsser, Meliana and Algarrobo) or locations locations (Alcàsser and Meliana). *Snedecor’s F* statistic and its significance (* *p* < 0.01, ** *p* < 0.001) is indicated in the table.

	Location		IL		Location x IL	
	3 L ^1^	2 L ^2^	3 L	2 L	3 L	2 L
Trait ^3^	*Snedecor’s F*					
FW (g)	163.89 **	2.23	37.24 **	25.55 **	2.71 **	1.76
FP (mm)	n.d. ^4^	27.45 **	n.d.	23.67 **	n.d.	1.75
FS	85.69 **	104.06 **	42.38 **	27.59 **	2.27 **	2.10 *
FL (mm)	260.46 **	19.7 **	50.69 **	32.99 **	2.16 **	1.30
FD (mm)	173.73 **	20.09 **	31.42 **	21.95 **	1.57	1.60
FA (cm^2^)	n.d.	4.68	n.d.	25.62 **	n.d.	1.64
FFP (%)	3.62	2.97	14.39 **	9.29 **	1.22	0.52
SSC (ºBrix)	186.17 **	51.84 **	9.27 **	5.74 **	1.79 *	1.20
FF (kg/cm^2^)	n.d.	31.06 **	n.d.	14.42 **	n.d.	1.83

^1^ Analysis on data from three locations. ^2^ Analysis on data from two locations. ^3^ Traits analyzed: FW (fruit weight), FP (fruit perimeter), FS (fruit shape, ratio length/diameter), FL (fruit length), FD (fruit diameter), FA (fruit area), FFP (fruit flesh proportion), SSC (soluble solid content) and FF (fruit firmness). ^4^ n.d.: no data.

**Table 3:** Introgression lines showing variation in rind color (RC) and the abscission layer formation (AL) with respect to PS in the three locations Alcàsser, Meliana and Algarrobo.

Trait	Alcàsser	Meliana	Algarrobo
Yellow background in rind	TRI03-2	TRI03-2	TRI03-2
Pale background in rind	TRI04-1	TRI04-1	
	TRI08-2	TRI08-2	TRI08-2
Abscission layer	TRI08-1	TRI08-1	n.d ^1^
	TRI08-2	TRI08-2	n.d.

^1^ n.d.: no data.

## Data Availability

Not applicable.

## Supplementary Materials

The following supporting information can be downloaded at: https://www.mdpi.com/article/10.3390/ijms241210099/s1.
