# Supplementary figures and images for: A Novel Introgression Line Library Derived from a Wild Melon Gives Insights into the Genetics of Melon Domestication, Uncovering New Genetic Variability Useful for Breeding
Source: Int J Mol Sci. 2023 Jun 14;24(12):10099. doi: 10.3390/ijms241210099 (PMC10298652; doi:10.3390/ijms241210099)

TRI01-1

TRI01-2

TRI01-3

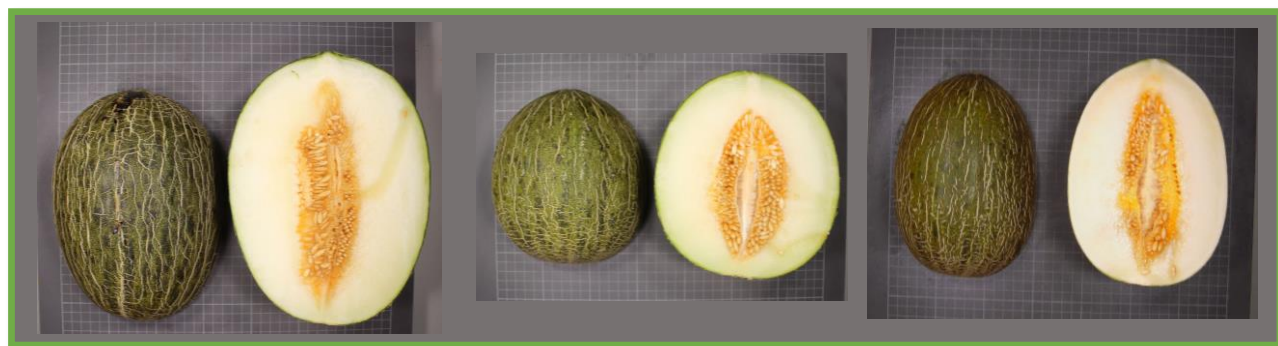

PS

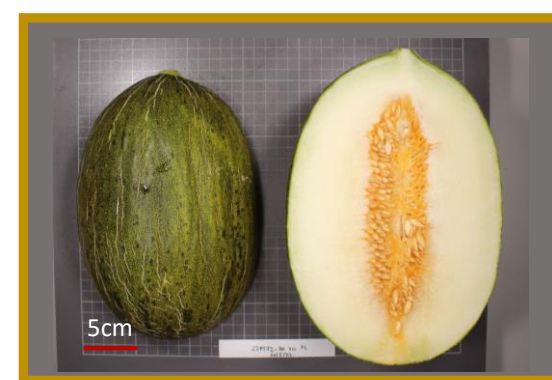

TRI02-1

TRI02-2

TRI02-3

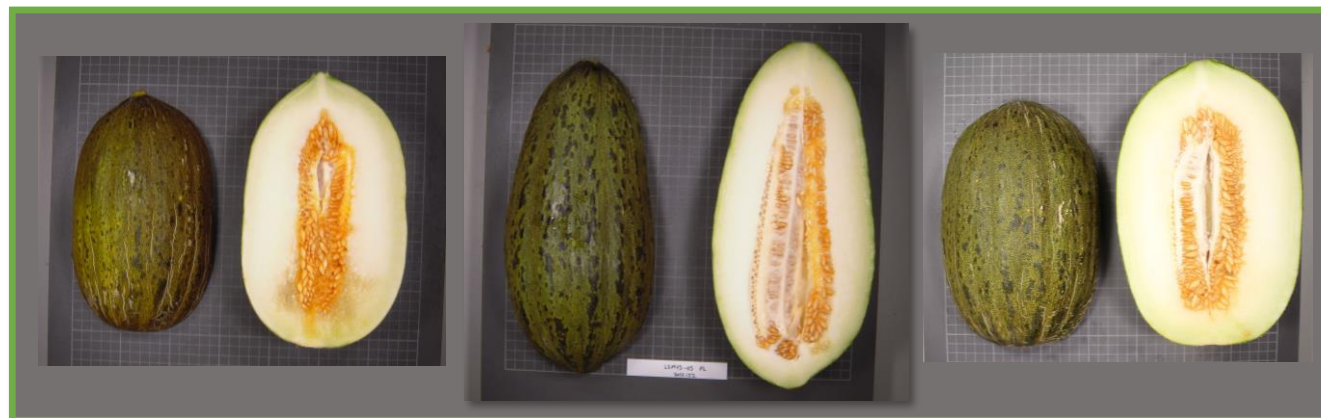

TRI03-1

TRI03-2

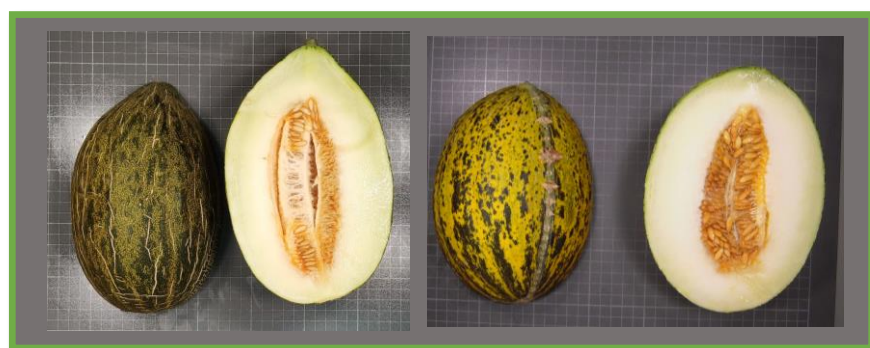

TRI04-1

TRI04-3

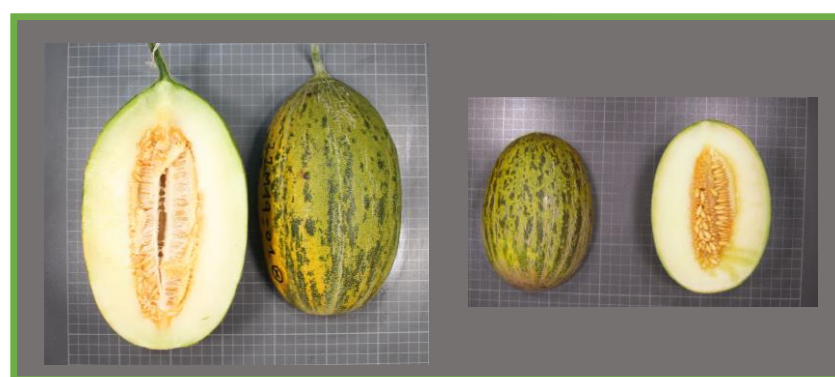

TRI05-2

TRI05-3

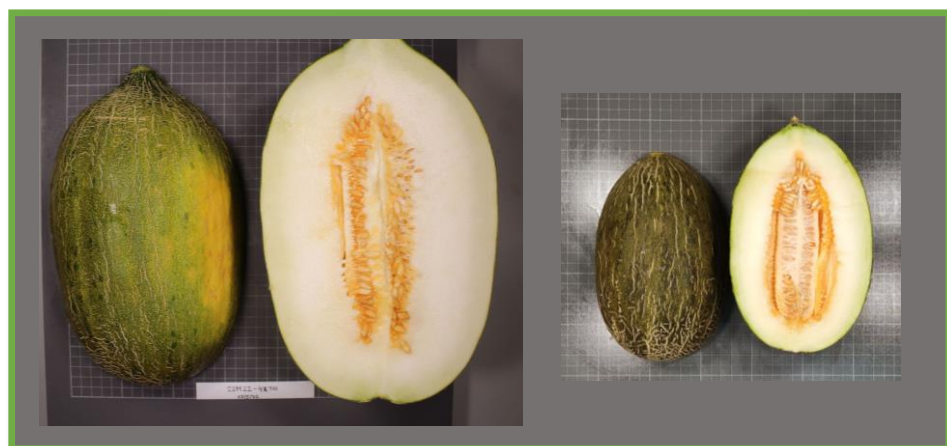

TRI06-1

TRI06-2

TRI06-4

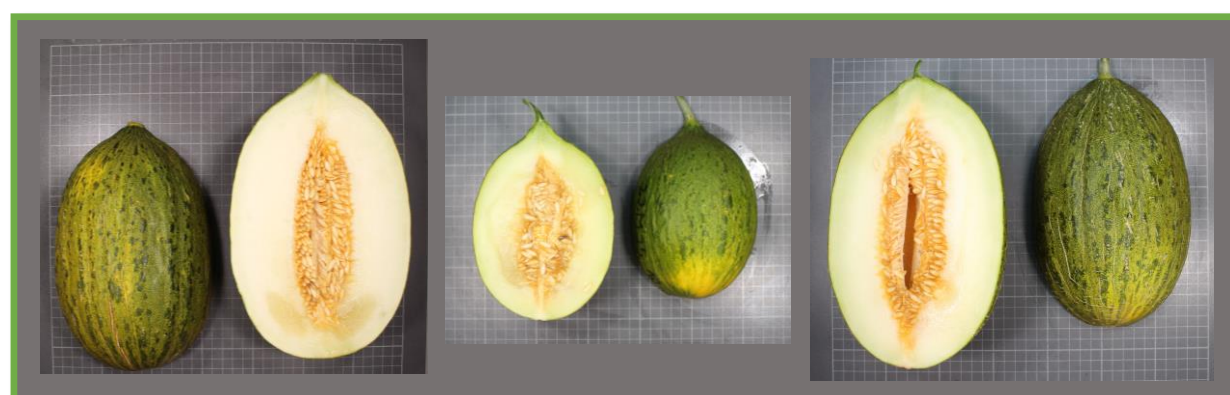

TRI07-2

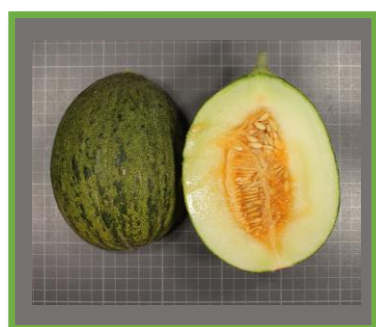

TRI08-1b

TRI08-2

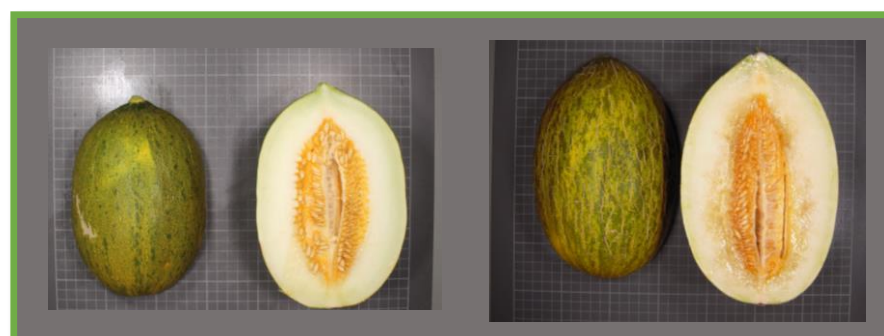

TRI10-0

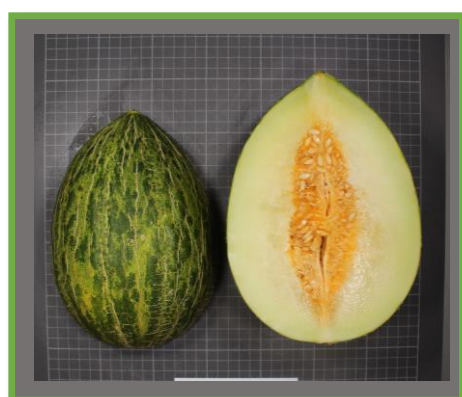

TRI11-2

TRI11-3

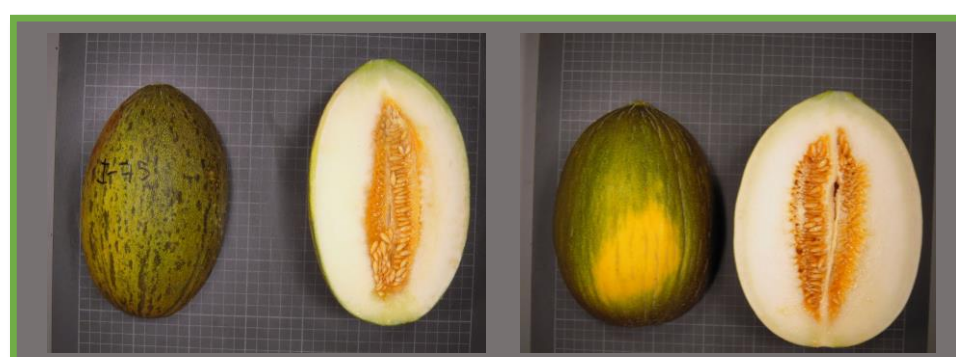

TRI12-1

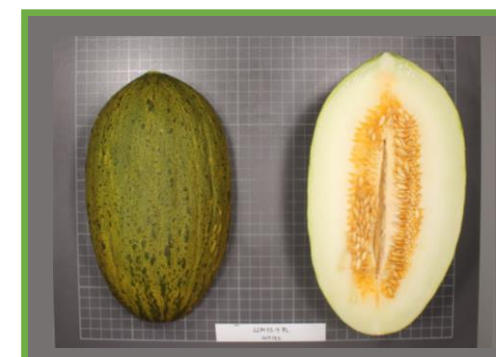

Supplement: Supplementary file 1 [file ijms-24-10099-s001.zip › Figure_S1. Representative images of the 22 ILs.pdf]
